# Sclerosing Pneumocytoma: A Ten-Year Experience at a Western Balkan University Hospital

**DOI:** 10.3390/medicina55020027

**Published:** 2019-01-25

**Authors:** Aleksandra Lovrenski, Milena Vasilijević, Milana Panjković, Dragana Tegeltija, Dejan Vučković, Ilija Baroš, Jovan Lovrenski

**Affiliations:** 1 Faculty of Medicine, University of Novi Sad, 21000 Novi Sad, Serbia; milena.vasilijevic@uns.ac.rs (M.V.); milana.panjkovic@mf.uns.ac.rs (M.P.); dragana.tegeltija@mf.uns.ac.rs (D.T.); dejan.vuckovic@mf.uns.ac.rs (D.V.); jovan.lovrenski@mf.uns.ac.rs (J.L.); 2 Department of Pathology, University Clinical Center of Republic of Srpska, 78000 Banja Luka, Republic of Srpska, Bosnia and Herzegovina; ilija.baros@med.unibl.org

**Keywords:** sclerosing pneumocytoma, histopathology, immunohistochemistry

## Abstract

*Background and objective:* Sclerosing pneumocytoma is a rare, benign tumor of the lung that represents a diagnostic challenge due to the diversity of pathohistological findings. The aim of this study was to present a 10-year experience with sclerosing pneumocytoma of a large center for the diagnosis and treatment of pulmonary diseases, and to emphasize differential diagnostic dilemmas as a potential source of errors. *Material and Methods:* This represents a retrospective study of six patients diagnosed and treated with sclerosing pneumocytoma in the 10-year period. The study analyzed various parameters, which are: Sex, age, symptoms, size and localization of the tumor, and its gross and histological features. *Results:* Sclerosing pneumocytoma was more frequently diagnosed in females (83.34%). The patients ranged in age from 38 to 61. Most of the patients (66.66%) were asymptomatic. Two patients underwent a video-assisted thoracoscopic surgery, two patients had a video-assisted minithoracotomy, and two patients underwent a thoracotomy in order to remove the tumor. The tumor was localized in the left lower lobe, in the right upper lobe, and in the right lower lobe in 50%, 33.34%, and 16.66% of patients, respectively. The tumor size ranged from 1 to 2.5 cm. A pathohistological examination of all six cases reported that all four major histological patterns were found in tissue sections: solid, papillary, sclerosing, and hemorrhagic. In all six cases, an immunohistochemical analysis showed positive expression of TTF-1 and panCK in surface epithelial cells, and TTF-1 positivity and panCK negativity in round stromal cells. *Conclusions:* Sclerosing pneumocytoma is a strictly histological diagnosis supported by clinical and radiological findings and corresponding immunohistochemical methods. Lung pathologists should always keep this tumor in mind, since its spectrum of differential diagnosis is wide, and therefore it can be an important diagnostic pitfall.

## 1. Introduction

One of the rare, benign tumors of the lung is sclerosing pneumocytoma (SP), which represents a pulmonary neoplasm with a complicated and an undefined histogenesis. This tumor was first described by Liebow and Hubbell over 60 years ago as an uncommon lesion with an uncertain origin. The original description by these two authors implied that this tumor originates from vascular endothelial cells; so, initially, this lesion was named sclerosing hemangioma [1]. Although its name implicated a vascular neoplasm, further studies have reported the possible pulmonary epithelium (pneumocyte type II) origin of this tumor. This conclusion has been strongly supported by immunohistochemical findings, and that is why alternative terms, such as pneumocytoma, sclerosing pneumocytoma, or papillary pneumocytoma, have been suggested [2]. In the newest World Health Organization (WHO) classification of lung and pleural tumors from 2015, it has been classified under the more convenient name pneumocytoma [3]. Although it is generally considered to be a benign tumor, it represents a diagnostic challenge due to its controversial etiology and biologic behavior, as well as the diversity of pathohistological findings.

The aim of this study was to present a 10-year experience with sclerosing pneumocytoma of a large center for the diagnosis and treatment of pulmonary diseases, and to emphasize differential diagnostic dilemmas as a potential source of errors.

## 2. Material and Methods

A retrospective, 10-year-period study included six patients diagnosed with sclerosing pneumocytoma. The study protocol was approved by the Ethics Committee of Institute for Pulmonary Diseases of Vojvodina (22 December 2016, No. 76-XV/6). All clinical data referring to age, symptoms, smoking status, surgical procedures, localization of the tumor, and diameter of the tumor were obtained from patients’ medical charts. The video-assisted thoracoscopic surgery (VATS), minithoracotomy, and thoracotomy were the surgical procedures performed in order to remove the tumor and to confirm the final diagnosis by histology. During surgery, a pathohistological examination on a frozen section specimen was performed in all six cases. The surgical specimen was embedded in a gel-like medium consisting of polyethilene glycol and polyvinyl alcohol (Bio-Optica, Milano, Italy) and placed on a metal tissue disc that was then frozen rapidly to about −20 to −30 °C. Afterwards, it was cut with the microtome of the cryostat on a histology 4-μm-thick slice. The section was picked up on a glass slide and stained with hematoxylin and eosin (H & E) stain. The preparation of the sample is much more rapid than a traditional histology technique. It takes about 10 min for whole procedure to be done; in comparison, the standard protocol for formalin-fixed, paraffin-embedded (FFPE) tissue takes much longer, since tissue should be fixed in formalin usually for at least for 12 h. However, the technical quality of the frozen sections is much lower, so a final pathohistological diagnosis should always be made on FFPE tissue. After taking a specimen for a frozen section analysis, more specimens from the tumor were taken for a standardized pathohistological analysis on FFPE tissue. All tissue sections were fixed by 10% neutral formalin, paraffin-embedded, sliced into 4-μm-thick sections, and stained with hematoxylin and eosin. The immunohistochemical analysis was performed by using TTF-1 and CK7 antibodies (Dako, Glostrup, Denmark), as well as additional staining for estrogen receptors (ER) and progesterone receptors (PR) (Dako, Glostrup, Denmark). All of the patients have been followed up for at least 5 years.

## 3. Results

During a 10-year period, we found six patients with SP: five females (88.34%) and one male (16.66%), Most of the patients (66.66%) were asymptomatic with changes discovered accidentally during routine medical examinations or while performing follow-up examinations of existing respiratory diseases. Within two patients (33.34%), the tumor presented with clinical symptoms. From data regarding medical charts, we discovered that two patients (33.34%) were smokers; one had a history of 25 pack/years, and the other a history of 10 pack/years. For the pathological examination of verified radiological masses, all patients underwent surgical procedures (Table 1).

**Figure 1:**
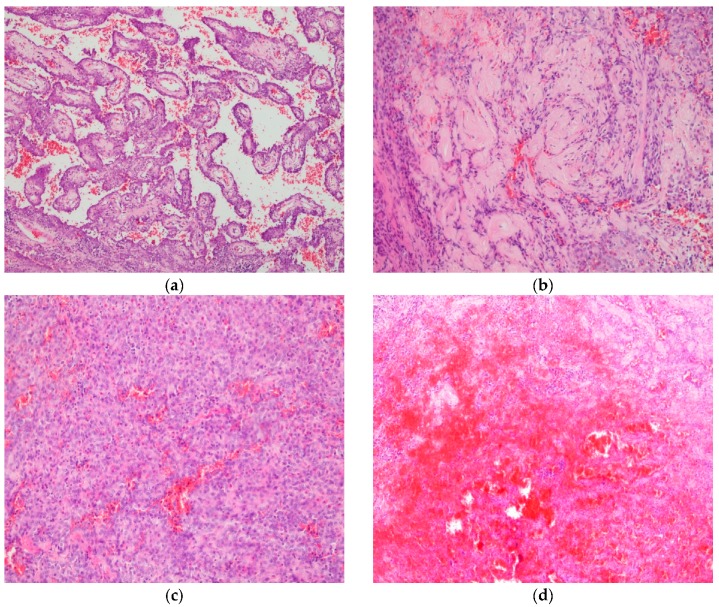
Histological patterns of sclerosing pneumocytoma (SP). (**a**). Papillary pattern, H & E, ×10; (**b**). Sclerosing pattern, H & E, ×20; (**c**). Solid pattern, H & E, ×20; (**d**). Hemorrhagic pattern, H & E, ×10.

In all six patients, during surgery, a pathohistological examination on frozen section specimens was performed. In three cases, pathologists set a diagnosis of SP; in one case, a diagnosis of clear cell tumor (so called “sugar tumor”) was made; and, in two cases, pathologists could not set an appropriate diagnosis on the frozen section specimen, so a final diagnosis was made on FFPE tissue. A pathohistological examination of all six cases on FFPE tissue revealed that this tumor was typically composed of two cellular components: surface epithelial cells that resemble type II alveolar pneumocytes, and round stromal cells. These cells were typically arranged in four major histological patterns: papillary, sclerosing, solid, and hemorrhagic. The papillary pattern was composed of complex papillae without a typical fibrovascular core. The papillae were lined with surface cells covering stroma with round cells (Figure 1a). The sclerosing pattern was formed by hyalinized collagen on the periphery of hemorrhagic areas, in papillae or in solid areas (Figure 1b). The solid pattern was characterized by sheets of uniform round cells or small tubules covered with surface cells, while large blood-filled spaces covered with epithelial cells that can contain hemosiderin deposits, foamy macrophages, or cholesterol fissures formed a hemorrhagic pattern (Figure 1c,d).

**Figure 2:**
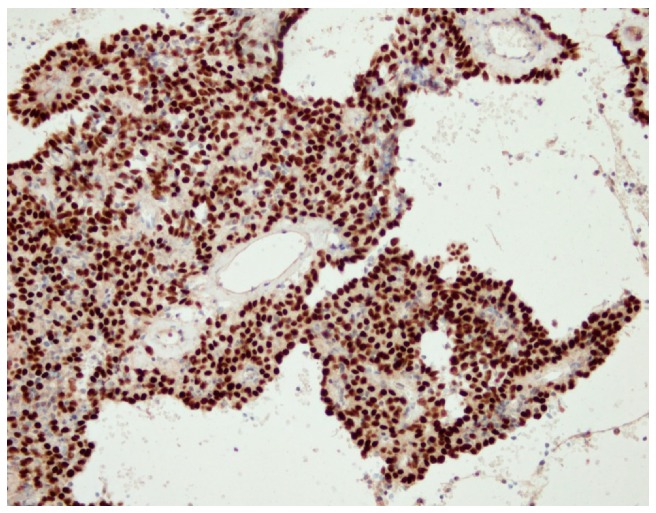
An immunohistochemical analysis showing positive expression of TTF-1 in both surface and round cells, ×40.

**Figure 3:**
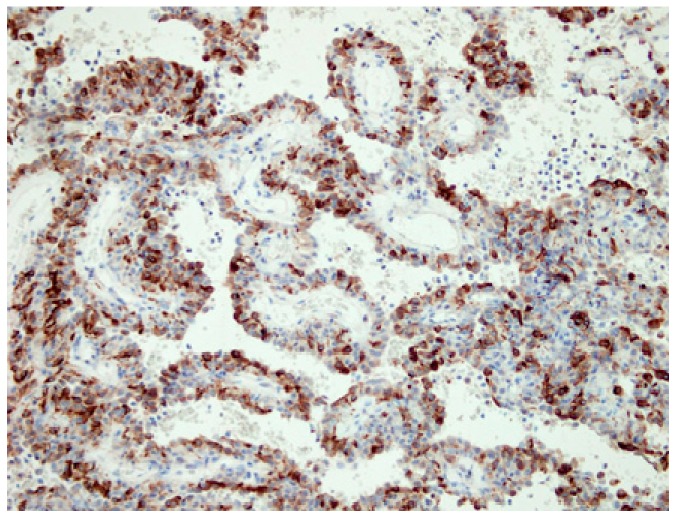
An immunohistochemical analysis showing positive expression of panCK in surface cells, but negative expression in round cells, ×40.

**Figure 4:**
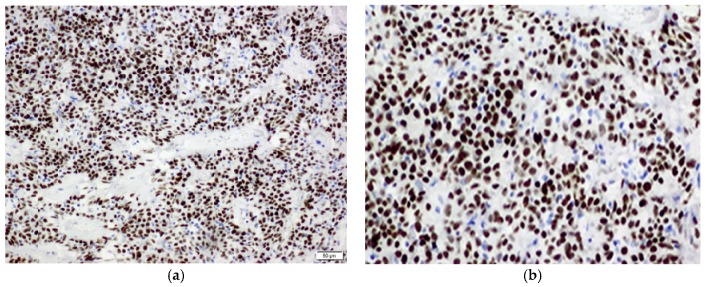
(**a**). An immunohistochemical analysis showing a positive reaction of the tumor cells on progesterone receptors, ×20; (**b**). An immunohistochemical analysis showing a positive reaction of the tumor cells on estrogen receptors, ×40.

In all six cases, an immunohistochemical analysis showed positive expression of TTF-1 and panCK in surface epithelial cells, and TTF-1 positivity and panCK negativity in round stromal cells (Figure 2 and Figure 3). Additionally, both ER and PR were studied immunohistochemically, with the following results: three patients were positive for PR and negative for ER (PR+/ER−) (50%), one patient was both PR and ER positive (PR+/ER+) (16.66%), and two patients were both PR and ER negative (PR−/ER−) (33.34%) (Figure 4a,b).

Patients were discharged from hospital 4–6 days after surgery. The presence of lymph node metastasis was not detected within these six cases, nor were any death outcomes reported during the 5-year follow-up period.

## 4. Discussion

Pulmonary sclerosing pneumocytoma is considered to be a rare benign neoplasm originating from type II pneumocytes.

Current studies have reported that the female population is affected more frequently, with the tumor generally occurring in the middle-aged population, with peak age incidence between the 4th and 7th decade [4,5]. This was in concordance with our study. Members of the female population have been frequently diagnosed with this tumor in eastern Asia, as the opposite of low incidence in western countries [4].

The majority of patients are asymptomatic, with the tumor being incidentally revealed as a soft-tissue mass on routine chest radiographs [6]. In our study, two-thirds of patients had no symptoms of the disease, while the most frequent symptom in clinical manifested disease was cough followed by fatigue, expectoration, and dyspnea. This history of unspecific symptoms can be explained by the fact that most frequent location of the tumor is within the lung parenchyma, and, as the mass grows with variable speed, the compression of adjacent lung tissue is usually manifested through the symptoms mentioned [4]. Although SP almost always involves the pulmonary parenchyma, rare cases of endobronchial presentation have been reported. The tumor in its endobronchial growth is usually presented as a polypoid mass with possible necrotic and hemorrhagic areas and edematous or ulcerative surrounding mucosa. Due to the growth, the tumor obstructs the lumen, which can lead to severe respiratory arrest. In order to reveal this rare location, bronchoscopy is required. If not diagnosed properly, this tumor can lead to postobstructive pneumonia [7]. Furthermore, bronchial cytology may be insufficient for the definitive diagnosis; therefore, a biopsy should be performed whenever possible [8,9]. Asymptomatic presentation can be related to extremely rare cases of SP with pleural dissemination. There has been only one report of this tumor with this kind of transfer described in the literature; however, the transfer mechanism remains unclear [10].

Two of the six patients in our research were smokers. In other available reports, the incidence of smokers and nonsmokers with diagnosed pulmonary SP was variable; however, none of these studies defined smoking as a risk factor or its influence on the development of this neoplasm [11].

Rare studies have reported that SP can be bilateral, which can be important in differential diagnosis from metastatic lung tumors [12,13]. In rare cases, SP can present as a cystic tumor when a radiological examination usually shows a well-defined oval lesion, hypoattenuate on contrast enhanced computed tomography (CT), and differently presented on (MRI) sequences. Microscopically, the cystic variant usually consists of a fibrous and partly hyalinized cystic wall, with tumor cells forming various histological patterns [14]. For obtaining a definitive diagnosis, various surgical procedures have been performed, so the possibility of malignancy can be excluded.

Surgical resection is currently widely used for treating SP. Xu and colleagues have suggested that surgical resection is curative for this tumor, and that no additional treatment after the surgery and no evidence of recurrence define the excellent prognosis of the disease [15]. Among the surgical techniques, video-assisted thoracoscopic surgery is the most common surgical procedure. Chen and colleagues have reported that 61.54% of all patients underwent this procedure [11]. In the present study, video-assisted thoracoscopic surgery, video-assisted minithoracotomy, and thoracotomy were equally performed.

Sclerosing pneumocytoma has no predilection for a particular lobe of the lung, which was confirmed by a study analyzing 28 cases with a definitive diagnosis. Among the peripheral lesions, five were located in the right upper lobe, four in the right middle lobe, six in the right lower lobe, nine in the left lower lobe, and three in the left upper lobe of the lung [16]. Our results are consistent with reports of an unspecific location of the tumor, with three cases of left lower lobe locating tumor, two cases of right upper lobe locating tumor, and one case of right lower lobe locating tumor.

The maximum diameters of SP in our patients varied from 1 to 2.5 cm. According to other studies, lesions range from 0.3 to 7 cm in greatest dimension [2,4,6]. Some authors have assumed that clinical symptoms can be evident due to enlargement and consequent pressure on adjacent lung parenchyma. However, other authors have disagreed with this assumption. Furthermore, one of the studies reported no relationship between tumor size and clinical appearance of the tumor [17]. Among the patients in our study, the smallest tumor (1 cm) presented with symptoms such as exhaustion and cough, while the biggest tumor (2.5 cm) manifested as dyspnea, cough, and expectoration. Patients with tumor measurements between the abovementioned diameters were asymptomatic.

Cytological findings of SP have not been helpful in the diagnosis of this tumor. Aspiration or imprint cytology may show polygonal cells, erythrocytes, and foamy macrophages, but the components of solid, papillary, sclerosing, and hemorrhagic patterns can also be occasionally found [18]. The cytological features of this tumor are suggestive of well-differentiated adenocarcinoma, which is considered to be the most important pitfall in the cytological differential diagnosis of SP. However, the presence of necrosis and multiple nuclei (more than three nuclei) within single cells are the features that strongly favor adenocarcinoma over SP. Furthermore, the two different cellular components and heterogeneous nature of the tumor helps SP to be distinguished from adenocarcinoma of the lung [6]. In our research, we did not perform a cytological analysis since all of the lesions were small in size and accessible for a surgical procedure. Also, all of the patients were in a good general condition without an increased risk for surgery. In three of the six patients included in this study, a diagnosis of SP was made on a frozen section; in one case, a diagnosis of clear cell tumor was set; and in the other two cases, pathologists could not say with certainty what the nature of the lesion was, so they left the definitive diagnosis to be set on FFPE tissue. Based on our experience, a diagnosis of this tumor can be a great challenge on frozen section specimens, taking into account the low technical quality of the frozen sections, the diversity of the histological image, and the fact that, for a frozen section analysis, usually one section from tumor tissue is taken. Within that particular section, all four patterns might not be found. For example, the presence of a hemorrhagic and a sclerotic component may lead to a misdiagnosis of pulmonary infarction at the organizing stage, while a finding of a papillary pattern can be interpreted as well-differentiated adenocarcinoma or a carcinoid tumor. The presence of a solid pattern is even more challenging and can result in a wide range of primary lung tumors, including clear cell tumor (so called “sugar tumor” due to intracytoplasmic glycogen or perivascular epithelioid cell tumor, PEComa), poorly differentiated adenocarcinoma and squamous cell carcinoma or large cell carcinoma, as well as undifferentiated metastatic carcinomas, metastatic melanoma, or even some types of lymphoma.

The diversity of pathohistological findings results in a wide range of differential diagnoses, which includes various benign conditions and benign and malignant tumors [19]. Among the benign conditions, besides the already mentioned pulmonary infarction, scar tissue, reactive or reparative changes in inflammation, post radiation changes, or changes as a result of a drug reaction can be misinterpreted as SP. While the sclerotic pattern in SP mimics scar tissue, reactive and reparative changes in response to inflammation or radiation or reaction to a drug result in hyperplasia of type II pneumocytes with a moderate degree of atypia and the formation of papillary structures, which imitate the papillary pattern of SP. Benign tumors of the lung in the differential diagnosis of SP are clear cell tumor, hamartoma, and hemangioma, with a variety of microscopic appearances. Tumor cells of clear cell tumor have a glycogen-rich abundant clear to eosinophilic granular cytoplasm with well-defined cell borders and show HMB45, MelanA, and S-100 positivity on immnuhohistochemical analysis. In hamartoma, apart from the presence of a bronchial epithelium, fragments of the mature cartilage can also be found, while in pulmonary hemangioma, true vascular spaces filled with blood and lined with CD31- and CD34-positive endothelial cells are present. Among the malignant neoplasms, lepidic and papillary lung adenocarcinoma can imitate SP; however, it does not exhibit the two-cell pattern of surface and round stromal cells, and a higher degree of cytologic atypia along with high mitotic activity is present. Carcinoid tumor can also be a diagnostic pitfall; however, a neuroendocrine appearance with rosette formation, “salt and pepper” chromatine, and positivity on CD56, Synaptophysin, and Chromogranin should lead to a proper diagnosis. Poorly differentiated non-small cell lung carcinoma, metastatic carcinomas, melanomas, or lymphomas may be excluded from differential diagnosis by identifying major histological patterns of the SP, its two cellular components, and mild cytologic atypia, while in difficult cases an immunohistochemical analysis is suggested [19,20].

A pathohistological examination is usually combined with an immunohistochemical analysis for the definitive diagnosis of SP. Previous studies have reported that both surface and round cells show a positive immunohistochemical reaction on certain antibodies, which facilitates the diagnosis of the tumor. A positive nuclear reaction on TTF-1 has been observed in both cellular components, while panCK has been expressed only in the cytoplasm of surface epithelial cells [19]. The fact that TTF-1 has been considered to be the characteristic antigen of alveolar epithelial cells strongly suggests the primitive respiratory epithelium origin of SP [21]. Based on the findings of higher incidence in females, the immunohistochemical analysis included both estrogen receptors and progesterone receptors. According to one study, most patients were positive for both ER and PR, suggesting a relationship between this tumor and female sex hormones [6]. Our study provided similar results, reporting all patients positive for TTF-1 in both types of cells and panCK in round cells, in three patients tumor cells showed positivity for PR, in one patient cells were positive for both ER and PR, while in two patients tumor cells were negative for both ER and PR.

The patients with SP have an excellent prognosis with no need for additional treatment after surgical resection. This tumor is generally considered to be a benign lesion, although potential malignant behavior is expressed through lymph node metastasis that was first reported in 1986 by Tanaka and colleagues [22]. Adachi and colleagues have demonstrated 18 cases with lymph node metastases, with a higher incidence in younger patients [23]. In another study, four cases of SP with metastases in hilar lymph nodes have been reported; however, after surgery, all patients were uneventful with no evidence of recurrence in a five-year period [24]. In the largest series of published cases, which included 100 patients, only one patient had a lymph node metastasis [19]. According to other studies, lymph node metastasis is more likely to occur in younger patients, with no influence on a patient’s prognosis [25,26]. The mechanism of the metastasizing of SP is still unclear. In our study, all patients showed no evidence of lymph node metastases.

## 5. Conclusions

Sclerosing pneumocytoma is a histological diagnosis supported by clinical and radiological findings and corresponding immunohistochemical methods. Lung pathologists should always keep this tumor in mind, since the spectrum of differential diagnoses is wide, and therefore it can be an important diagnostic pitfall.

## Figures and Tables

**Table 1:** Characteristics of patients with sclerosing pneumocytoma.

	Patient 1	Patient 2	Patient 3	Patient 4	Patient 5	Patient 6
Gender	F	F	F	F	F	M
Age	45	61	60	38	53	47
Smoking	Yes	No	No	No	No	Yes
Clinical symptoms	Without symptoms	Fatigue, cough	Without symptoms	Without symptoms	Dyspnea, cough, expectoration	Without symptoms
Reason for initial chest X-ray	Routine medical examination	Respiratory symptoms	Routine medical examination	Follow-up examination of asthma	Respiratory symptoms	Follow-up examination of COPD
Tumor localization	RLL	LLL	LLL	RUL	LLL	RUL
Tumor size	1.7 cm	1 cm	2 cm	1.5 cm	2.5 cm	1.8 cm
Pathohistological finding	All four patterns present	All four patterns present	All four patterns present	All four patterns present	All four patterns present	All four patterns present
Immunohistochemical finding	Surface cells: TTF1+, PanCK+ Round cells: TTF1+, Pan CK− All cells: PR+/ER−	Surface cells: TTF1+, PanCK+ Round cells: TTF1+, Pan CK- All cells: PR+/ER−	Surface cells: TTF1+, PanCK+ Round cells: TTF1+, Pan CK- All cells: PR+/ER+	Surface cells: TTF1+, PanCK+ Round cells: TTF1+, Pan CK− All cells: PR+/ER−	Surface cells: TTF1+, PanCK+ Round cells: TTF1+, Pan CK− All cells: PR−/ER−	Surface cells: TTF1+, PanCK+ Round cells: TTF1+, Pan CK− All cells: PR−/ER−
Treatment	Video-assisted minithoracotomy	Thoracotomy	VATS	Video-assisted minithoracotomy	Thoracotomy	VATS
Postoperative hospital stay (days)	4	6	6	5	4	4
5-year follow-up	Uneventful after surgery	Uneventful after surgery	Uneventful after surgery	Uneventful after surgery	Uneventful after surgery	Uneventful after surgery

COPD, chronic obstructive pulmonary disease; RLL, right lower lobe; LLL, left lower lobe; RUL, right upper lobe; VATS, video-assisted thoracoscopic surgery; TTF-1, Thyroid Transcription Factor-1; PanCK, Pan-Cytokeratin; PR, Progesterone; ER, Estrogen.
